# Compromise or Alternation? Experimental Evidence on Coordination Under Payoff Asymmetry

**DOI:** 10.3390/bs16030403

**Published:** 2026-03-10

**Authors:** Yilin Lv, Huiru Wang, Liyan Wu, Jie Zheng

**Affiliations:** Center for Economic Research, Shandong University, Jinan 250100, China; yilin.academic@mail.sdu.edu.cn (Y.L.); huiru.academic@mail.sdu.edu.cn (H.W.); lywu2025@sdu.edu.cn (L.W.)

**Keywords:** compromise, payoff asymmetry, alternation, battle-of-the-sexes, laboratory experiment

## Abstract

While the previous literature identifies the compromise option as a potential coordination device, it remains unclear how varying degrees of payoff asymmetry affect its adoption. This study experimentally examines coordination behavior in a repeated battle-of-the-sexes (BOS) game with a compromise option across three distinct levels of payoff asymmetry. We implement three between-subjects treatments that vary the degree of payoff asymmetry in the original BOS game while fixing the payoff for the compromise option. Under a hybrid matching protocol, we find that when the payoff asymmetry is higher, more groups coordinate on the compromise option. While payoff asymmetry initially reduces the coordination rate, repeated interaction mitigates this effect through learning. The alternation strategy is shown to be more efficient than the compromise one, though both enhance fairness. Our results reveal how the degree of payoff asymmetry influences subjects’ strategy adoption between compromise and alternation.

## 1. Introduction

Coordination problems are prevalent in economics, and conflicts of interest make coordination particularly difficult. When players hold divergent preferences, coordination is often essential for achieving mutual gains. However, there may exist multiple coordinating strategies, and the distribution of payoffs among players across these different coordination strategies can vary, potentially causing fairness concerns among players. A typical framework to study such coordination problems with conflict of interest is the battle-of-the-sexes (BOS) game, in which a couple shares the same view in terms of preferring coordinated outcomes to uncoordinated ones but differs in their preferences across the coordinated outcomes: the husband favors watching a football match together, while the wife likes to see a ballet performance together. When husband and wife choose the same activity, a successful coordination occurs, yielding positive payoffs for both of them, yet one player’s payoff is always greater than the other’s.

When such conflicts of interest between the players cannot be resolved by simply choosing the same available options, they may seek an alternative solution that serves as a compromise for both sides. In contrast to the pursuit of an efficiency goal, the compromise option as a coordination device prioritizes fairness. Consider international climate negotiations as an example: developed nations typically advocate for stringent emissions targets, whereas developing countries emphasize the right to develop their economies. The resulting agreement often constitutes a compromise: each side makes concessions to reach a consensus, accepting a less efficient outcome in exchange for greater perceived equity.

While compromise is common practice for resolving coordination issues, under the scenario of repeated interactions, players may devise alternative solutions to dynamically coordinate. An alternation strategy can emerge, in which players take turns to choose their high-payoff options. This approach enhances both efficiency and fairness over time, but requires coordination over the time dimension. One example of alternation as a coordination device is firms bidding for repeated government contracts. While a one-shot procurement might lead to aggressive bidding, a long-term horizon enables firms to dynamically coordinate by taking turns to submit a low-level bid. Thus, although only one firm profits in any given round, all participants enjoy the gains over the time horizon.

The issue of payoff asymmetry across players is central to understanding why coordination may fail despite the presence of focal points. Since a coordination problem arises when a game has multiple equilibria, seminal works by Schelling (1960), as well as Harsanyi and Selten (1988), emphasize that individuals rely on focal points, salient cues, or expectations of others’ actions to achieve coordination. However, payoff asymmetry can weaken the effectiveness of focal points in coordination (Parravano & Poulsen, 2015) and cause players to focus more on their own gains than on the team’s gains (Faillo et al., 2013). Since players adjust their coordination strategies when payoff structures change (Camerer & Hogarth, 1999), coordination may become even harder when there exist multiple coordination devices under conflicts of interest.

When payoffs for the one-shot coordinated outcomes are highly asymmetric, conditional on a successful coordination, the high-payoff player may have a strong incentive to adhere to their own preference, while the low-payoff player may want to switch to a different coordinated outcome that he or she prefers. Such a payoff asymmetry not only reduces the rate of initial successful coordination but can also undermine the maintenance of an established coordination. For example, in the battle-of-the-sexes game, payoff asymmetry usually leads to lower coordination rates than those observed in pure coordination games (Cooper et al., 1989; Leng et al., 2023), providing supporting experimental evidence for our study.

Our paper extends the framework of He and Wu (2020), who introduced a compromise option into the battle-of-the-sexes (BOS) game. While they examined the effectiveness of this option under different matching protocols (fixed vs. random), we maintain a fixed matching protocol to identify another driver of behavior: the degree of payoff asymmetry. He and Wu (2020) demonstrate that a compromise option—characterized by symmetric but inefficient payoffs—often serves as a salient focal point. It mitigates the coordination risk associated with the two efficient but asymmetric equilibria. However, a crucial question remains: how does the intensity of inequality in the efficient outcomes reshape this trade-off? While the compromise option offers a “fair” static solution, players in a repeated setting may arguably achieve higher efficiency through a dynamic alternation strategy. It is unclear whether the choice between the simple compromise strategy and the more profitable alternation strategy is sensitive to the degree of the payoff asymmetry.

To address this, we designed a laboratory experiment to investigate how the degree of payoff asymmetry affects the adoption of these two coordination strategies. Following the 3×3 BOS game structure with a compromise option employed by He and Wu (2020), we implement a fixed-partner design over 20 rounds. Crucially, unlike previous studies that held asymmetry constant, we designed three different payoff asymmetry structures: (250, 50) as the highest asymmetry, (220, 80) as moderate asymmetry, and (180, 120) as the lowest asymmetry. This design allows us to identify whether and how the severity of conflict in the efficient equilibria drives players towards or away from the compromise option.

We find that the degree of payoff asymmetry acts as a critical determinant of both the likelihood and the mode of coordination. Specifically, the highest asymmetry results in the lowest overall coordination rate and induces subjects to choose the compromise option. In terms of strategy adoption, subjects in the lowest asymmetry condition are less likely to adopt the compromise strategy compared to those in the high and moderate asymmetry levels. Nevertheless, alternation is the most frequently adopted strategy in all treatments. Based on these results, we focus on the dynamics of alternation strategy and find that higher payoff asymmetry promotes learning, which increases the speed at which alternation is adopted across phases. Moreover, we find that “double experience”, when both subjects have prior experience with alternation, increases the likelihood that they adopt alternation. Finally, while the compromise and alternation strategies enhance fairness, we document a negative correlation between payoff asymmetry and average earnings, indicating that structural inequality imposes an efficiency cost.

This paper makes two main contributions. First, we advance the experimental literature on coordination by identifying the degree of payoff asymmetry as a critical determinant of strategy selection. Our design identifies the intensity of inequality as a mechanism that modulates the trade-off between risk dominance and payoff maximization. We show that the degree of payoff asymmetry shifts the relative likelihood of coordinating on the secure compromise option versus the efficient alternation strategy. This provides a more detailed explanation for the differences in coordination behavior observed in asymmetric conflicts. Second, we provide experimental evidence on the efficiency loss of structural inequality. Our results demonstrate that high payoff asymmetry creates a “fairness trap”: it increases the tendency to settle for the sub-optimal compromise option to mitigate conflict, thereby eroding a more efficient alternation strategy. This finding implies that excessive structural inequality imposes an intrinsic efficiency loss by hindering optimal long-term cooperative conventions. By documenting how structural parameters shape welfare outcomes, our study provides experimental evidence and theoretical support for countries to prevent excessive income inequality. That said, experimental economics helps bridge the gap between economic theory and real economic life (Levine & Zheng, 2015).

The remainder of this paper is organized as follows. Section 2 reviews the literature. Section 3 describes the experimental design and implementation. Section 4 presents the results, and Section 5 concludes the paper.

## 2. Literature Review

Since coordination can generate higher payoffs, players have strong incentives to coordinate their behavior (Crawford & Haller, 1990; Dong et al., 2024; Kandori et al., 1993; Young, 1996). However, coordination is often difficult to achieve in practice. On the one hand, in the absence of salient cues or common knowledge, individuals may find it hard to predict their partners’ actions, leading to coordination failure (Crawford et al., 2008). On the other hand, when payoffs are asymmetric, concerns about fairness can further hinder successful coordination.

The previous literature indicates that payoff asymmetry affects coordination. Unequal payoffs reduce coordination, as players tend to favor outcomes that are fairer but less efficient (Clark et al., 2001; Isoni et al., 2019). Even minor differences in payoffs can generate strategic uncertainty, eroding players’ confidence in converging on a common equilibrium and consequently hindering coordination (Crawford & Haller, 1990). Payoff asymmetry not only changes the incentives for players but also affects their expectations of others’ behavior. Neumann et al. (2023) conduct an experiment analyzing the relationship between an individual’s choices, their expectations, and their risk attitudes. They find that expectations explain individuals’ strategy selection. When there is a gap in payoffs, players must weigh their own interests against their expectations of others’ actions. In such cases, beliefs about others’ risk preferences play a crucial role in determining the coordination outcome (Crawford & Iriberri, 2007).

The effect of payoff asymmetry is also clear in repeated games. Sonsino and Sirota (2003) and Crawford et al. (2008) propose that coordination is less stable under asymmetric payoffs. Lau and Mui (2008) introduce the concept of “conflict level” in repeated battle-of-the-sexes games, and they find that higher conflict levels delay equilibrium because players behave more self-interestedly in the early rounds. Cason et al. (2013) show in laboratory experiments that learning effects in repeated rounds promote the adoption of alternation strategies, although their impact depends on the degree of conflict of interest. Arifovic and Ledyard (2018) and Elten and Penczynski (2020) argue that payoff asymmetry decreases the percentage of those alternating between the two pure-strategy Nash equilibria. Lien and Zheng (2019) introduce a voluntary membership fee and a penalty into the Prisoner’s Dilemma game and find that changes in the payoff structure influence cooperative behavior. Eckel and Wilson (2007) show that in a coordination game with a risky payoff-dominant and a safe risk-dominant equilibrium, observing an agent choose the payoff-dominant option increases coordination on it, indicating that both payoff structure and others’ behavior shape equilibrium selection. Therefore, we argue that the payoff structure has a profound impact on equilibrium outcomes.

To enhance coordination in the BOS game, some studies have introduced a compromise option. The compromise option has two main advantages: First, players can obtain higher payoffs than the unfavorable option and reduce payoff uncertainty. Second, the payoff gap is reduced, improving the fairness of coordination. In the experiment by Jackson and Xing (2014), one compromise option with equal payoffs but lower total payoffs and two asymmetric options were provided. They found that in one-shot games, most players chose the compromise option, even though it was inefficient. Similarly, Bett et al. (2016) conduct an experiment using a one-shot battle-of-the-sexes game with a third option, revealing that subjects tend to choose a symmetric, but strictly dominated option to avoid coordination failure. However, in some cases, the compromise option may hinder rather than facilitate coordination. As a result, offering a compromise may reduce the likelihood of reaching an efficient outcome (Libich & Nguyen, 2022; Sunstein & Thaler, 2008). In addition, Arjona et al. (2022) use the pie game and manipulate salience by increasing non-salient slices, finding that increasing label salience counteracts the negative impact of conflicts of interest and improves coordination.

In one-shot games, subjects tend to choose the equal-payoff compromise option to avoid coordination failure (Bett et al., 2016; Isoni et al., 2019). In the long run, subjects can make more efficient choices based on their previous interaction experiences, such as alternating between the two asymmetric pure Nash equilibria. Alternation strategy yields the highest total payoff that can be achieved in a repeated-game setting. Therefore, subjects’ strategies are not always obvious in repeated games. Blume and Gneezy (2000) argue that even without common knowledge, subjects can learn and achieve coordination in repeated games. Cason and Mui (2014) confirm that repetition and communication facilitate coordination. Ye et al. (2020) further show that gradually increasing contribution stakes over time—instead of requiring high contributions immediately—improves coordination in multiperiod laboratory experiments, suggesting that gradualism can promote efficient strategy adoption. Bhaskar (2000) demonstrates that in repeated symmetric games, focusing on symmetric equilibria can achieve efficiency along a single path, especially in games with finite repetitions or when players have moderate patience. This suggests that in repeated BOS games, considering symmetry can help participants reach efficient outcomes. He and Wu (2020) defined the equilibrium that increases payoff symmetry at the expense of efficiency as the compromise option, and found that while the compromise option improves coordination rates in one-shot games, it does not enhance coordination in repeated games. Instead, it delays the adoption of the alternation strategy and leads to a reduction in total payoffs.

In addition, there are several factors that influence successful coordination, such as communication, emotions, and leadership. Communication is largely informative. Hu et al. (2020) find that pre-play communication significantly increases payoffs and coordination probability while reducing miscoordination. Over the past fifteen years, some specific research has emphasized the importance of interpersonal emotions in conveying intentions, goals, and desires (Ferracci et al., 2022). Jiang and Pan (2023) conduct an online experiment to investigate how information about counterparts’ emotions influences aggressive or accommodating decisions and coordination outcomes in battle-of-the-sexes games. Their results show that providing emotional information significantly enhances coordination rates. Leaders can also influence coordination behaviors. The effectiveness of leadership depends on leader selection procedures and incentive structures. He and Zheng (2024) and Xu et al. (2025) provide experimental evidence that voluntary leadership from a randomly selected candidate promotes coordination through the signaling effect of leadership and mitigates concerns arising from incomplete information.

In summary, the existing literature identifies several factors that affect coordination, including the payoff structure, compromise options, repeated games (Wang & Zheng, 2015), and communication. Our study complements He and Wu (2020). As individuals respond differently to increases in inequality (Aoyagi et al., 2022), we set up three different payoff structures in the experiment. This study examines whether subjects’ strategies vary with the degree of payoff asymmetry and provides new insights into the impact of payoff asymmetry on coordination and decision-making behavior.

## 3. Experimental Design

### 3.1. Treatments

This section describes the experimental design with treatment information, the decision-making tasks with payoff structure, and the flowchart of conducting the experiment.

In the experiment, we implement a 3×3 battle-of-the-sexes game with a compromise option, following the design framework of He and Wu (2020). Each game features two payoff-asymmetric pure-strategy Nash equilibria, (A, A) and (B, B), and one payoff-symmetric pure-strategy Nash equilibrium, (C, C).^1^ When players choose different actions, both receive a payoff of zero. The row player (Player 1) prefers coordinating on (A, A), whereas the column player (Player 2) prefers coordinating on (B, B). The compromise outcome (C, C) yields an equal payoff of 100 for both players. Importantly, while (C, C) is the only outcome providing symmetric payoffs, it reduces efficiency compared to the asymmetric equilibria, for which the sum of payoffs is 300. We therefore refer to action C as the “compromise” option, as it represents a fair but inefficient coordination outcome.

To investigate how varying degrees of payoff asymmetry influence the trade-off between fairness and efficiency, as well as coordination outcomes in the presence of a compromise option, we change the payoffs at the asymmetric equilibria across three between-subjects treatments while keeping the compromise payoff fixed at (100, 100):

1. HA (High Asymmetry). Treatment *HA* features the most pronounced conflict of interest between the two players with payoffs of (250, 50) for (A, A) and (50, 250) for (B, B).
2. MA (Moderate Asymmetry). Treatment *MA* implements intermediate asymmetry with payoffs of (220, 80) for (A, A) and (80, 220) for (B, B).
3. LA (Low Asymmetry). Treatment *LA* represents minimal asymmetry with payoffs of (180, 120) for (A, A) and (120, 180) for (B, B).

**Figure 1:**
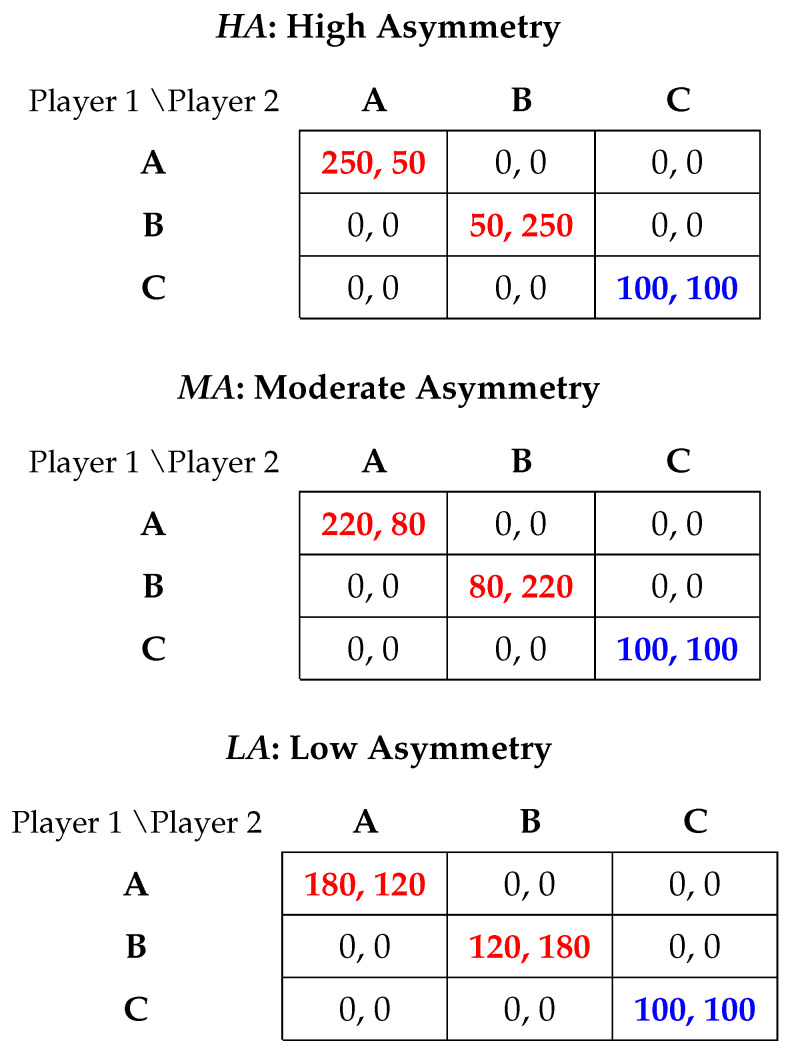
Payoff structures. Notes: Numbers in red indicate payoff-asymmetric pure-strategy Nash equilibria payoffs and numbers in blue indicate payoff-symmetric pure-strategy Nash equilibria payoffs.

The payoff matrices for the three treatments are shown in Figure 1, with all payoffs denoted in experimental tokens.

Then, we investigate the decision-making strategy of participants in repeated interactions. Each experimental session consisted of a practice phase followed by three formal game phases, with identical game settings applied throughout all phases. In each phase, participants were randomly matched into pairs and randomly assigned the roles of Player 1 or Player 2 within each pair. These pairings and roles then remained fixed for the entire subsequent 20 rounds. This matching protocol creates a fixed partnership within each phase, allowing participants to rely on previous interactions within the same pair to build future strategies. At the end of each round, participants received feedback on their partner’s action and their own payoff.

In all treatments, subjects receive feedback on their partner’s choice and their payoff after each round. Our experimental design differs from He and Wu (2020) in two important aspects. First, while their study examined coordination behavior under a single high-asymmetry payoff structure (250, 50 and 50, 250), we vary the degree of payoff asymmetry across three treatments while keeping the compromise payoff fixed at (100, 100). This setting enables us to investigate how the severity of conflict of interest influences the trade-off between fair compromise and efficient coordination across different levels of inequality. Second, He and Wu (2020) implemented either pure fixed matching or pure random matching across treatments. In the fixed matching condition, subjects formed long-term partnerships and could rely on previous interactions to build future strategies, which facilitates the development of history-dependent behaviors. Under random matching, subjects were paired with different partners each round and could not form lasting partnerships, effectively creating a series of one-shot decisions. In contrast, we employ a hybrid approach: subjects maintain fixed partnerships within each 20-round phase but are randomly re-matched with role switching between phases. Our design allows observation of learning within stable partnerships while generating multiple independent observations across different pairs.

**Figure 2:**
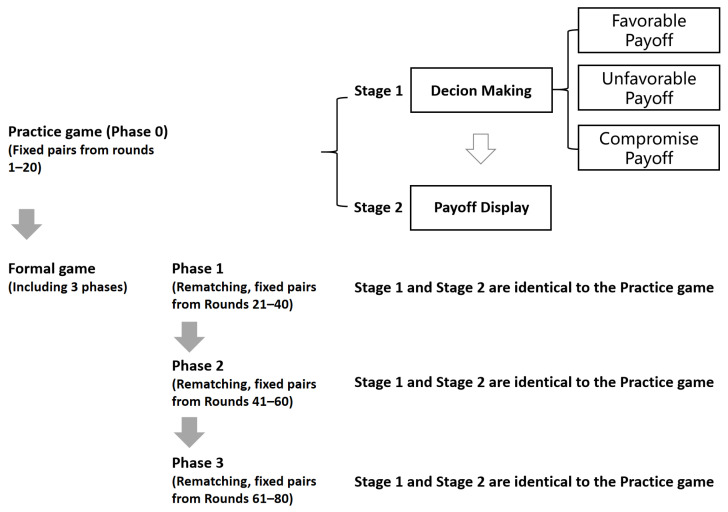
Flowchart of the experiment.

The whole experiment is summarized in Figure 2.

### 3.2. Hypotheses

We test three hypotheses in the experiment. First, the compromise option serves as an effective focal point for coordination by offering equal payoffs to both players. When the payoff gap between the unfavorable payoff in the asymmetric equilibrium and the compromise payoff is negative (50 vs. 100 and 80 vs. 100), we expect subjects to be more motivated to adopt the compromise strategy. Moreover, since the compromise option reduces inequality in payoff distribution, it improves coordination in the battle-of-the-sexes game under asymmetric payoffs. However, when the lower payoff in the asymmetric equilibrium exceeds the compromise payoff (120 vs. 100), subjects may prefer the alternation strategy to maximize their payoffs. We thus propose our first hypothesis, as follows.

**Hypothesis** **1.** *Subjects’ likelihood of adopting the compromise strategy is positively correlated with payoff asymmetry.*

Second, for each treatment, we conducted four phases of the games,^2^ each consisting of 20 rounds. He and Wu (2020) find that the alternation strategy improves efficiency in repeated games. However, sustaining the tacit coordination required for alternation imposes high initial strategic uncertainty. We hypothesize that accumulated experience mitigates this uncertainty through a dual mechanism: it not only increases the likelihood of adopting the efficiency-enhancing alternation strategy but also accelerates the convergence speed, allowing pairs to establish stable coordination in fewer rounds.

**Hypothesis** **2.** *Alternation experience in prior play increases the likelihood that subjects adopt the alternation strategy, and a higher degree of payoff asymmetry increases the speed of alternation strategy adoption.*

Introducing the compromise option increases the success rate of coordination. Both successful alternation and compromise lead to fairer outcomes compared to coordination failure. Alternation ensures that subjects take turns to receive the higher payoff, while compromise guarantees an equal payoff, thereby enhancing the overall fairness of outcomes whenever either form of coordination is achieved. The last hypothesis is thus regarding the welfare consequences of the coordination strategies.

**Hypothesis** **3.** *Both alternation and compromise strategies can enhance the fairness of payoff distribution, while the former achieves a more efficient outcome.*

### 3.3. Procedures

The experiment was conducted online in April 2020 using the z-Tree software (version 4.1.6) (Fischbacher, 2007) due to pandemic-related restrictions. A total of 96 subjects were recruited from Tsinghua University, representing diverse academic disciplines. The study followed standard ethical protocols and was approved by the Institutional Review Board (IRB) prior to data collection. All subjects signed the consent form and agreed to participate in the experiment. Table 1 presents the number of subjects, number of independent matching groups, and number of sessions in each treatment.

The experimental instructions were administered to participants in electronic format. The participants were mandated to complete the tasks independently in a secluded and quiet environment to prevent any external disruptions. To ensure procedural clarity and experimental control, the experimenter conducted an online meeting. During this meeting, the instructions were reviewed in detail, and a key measure was implemented: all participants were assigned anonymized and randomized codes that replaced their true identities. This protocol was designed to effectively eliminate the possibility of communication between participants regarding the experimental tasks. Furthermore, any subsequent queries from participants were addressed through private and one-on-one communication channels to maintain the integrity of the independent decision-making environment.

Each experimental session lasted approximately 35 min. The practice phase (Phase 0) consisted of 20 rounds without monetary consequences. In the main experiment, which included 60 rounds, earnings were accumulated across all rounds and converted at the rate of 1 token = CNY 0.0025. The average payment received ranged from CNY 18.80 to 19.98 per subject (including a CNY 5 show-up fee).

## 4. Results

In this section, we report the results of our experiments. First, we compare subjects’ coordination outcomes and strategies across treatments. Second, we examine the effect of the experience on the adoption of alternation strategies. Third, we analyze the efficiency and fairness under different strategies.

### 4.1. Subjects’ Coordination Strategy Across Treatments

#### 4.1.1. The Effect of Payoff Asymmetry on Coordination

In this section, we investigate the effect of payoff asymmetry on coordination. An outcome is classified as coordination if both subjects in a pair obtain positive payoffs ((A, A), (B, B), or (C, C)). The average coordination rate in each treatment is defined as the proportion of the 60 rounds (across the three phases) in which both subjects in a pair select one of these outcomes. Coordination consists of both compromise (C, C) and non-compromise ((A, A) or (B, B)) outcomes. We further analyze the impact of the compromise option as a reference point on coordination. The average compromise rate in each treatment is defined as the proportion of the 60 rounds (across the three phases) in which both subjects in a pair choose (C, C). The non-compromise coordination rate in each treatment is defined as the proportion of the 60 rounds (across the three phases) in which both subjects in a pair choose (A, A) or (B, B).

**Figure 3:**
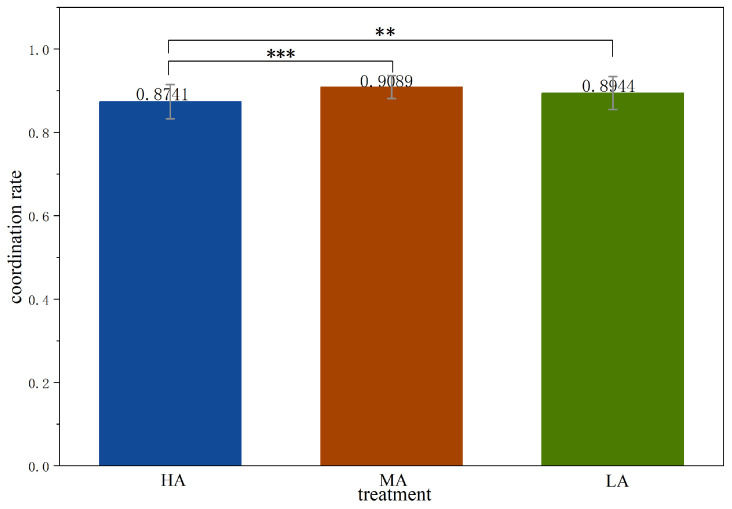
Average coordination rate across treatments. Notes: *** p<0.01, ** p<0.05.

As shown in Figure 3,^3^ the overall coordination rate is lowest in *HA* (0.8741), compared with 0.9089 in *MA* and 0.8944 in *LA*. Coordination in *HA* is significantly lower than in *MA* and and *LA* (Mann–Whitney test, *HA* vs. *MA*, *p* = 0.0005; *HA* vs. *LA*, *p* = 0.0468). The difference between *MA* and *LA* is not statistically significant (Mann–Whitney test, *MA* vs. *LA*, *p* = 0.1456). Under the highest payoff asymmetry, the favorable option offers the highest payoff, giving each subject an incentive to choose it, thereby leading to coordination failure. When payoff asymmetry is moderate or low, the favorable option does not provide sufficient incentives for subjects to risk coordination failure, so the unfavorable and compromise options are also acceptable choices. These findings are summarized in the following result.

**Result** **1.** *The coordination outcomes are less frequent in treatment HA than in treatments MA and LA.*

**Figure 4:**
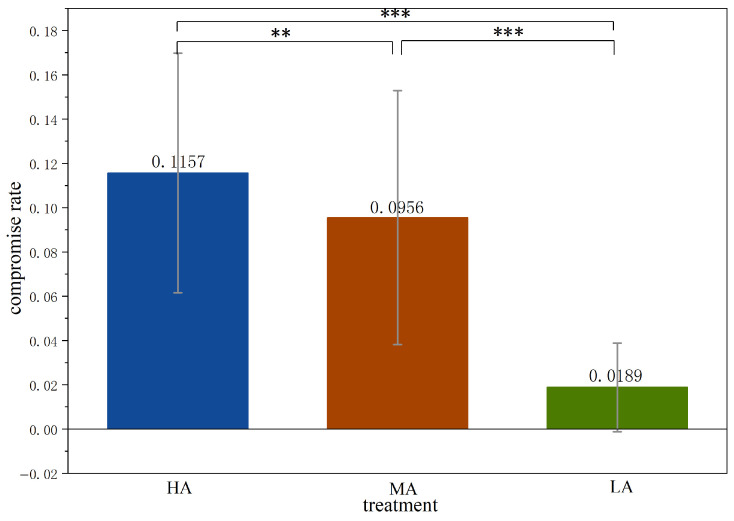
Average compromise rate across treatments. Notes: *** p<0.01, ** p<0.05.

As shown in Figure 4, the compromise rate is 0.1157 in *HA*, higher than in other treatments (0.0956 in *MA*, and 0.0189 in *LA*). The compromise rate in *HA* is significantly higher than in other treatments (Mann-Whitney test, *HA* vs. *MA*, *p* = 0.0404; *HA* vs. *LA*, *p* < 0.0001). Furthermore, the compromise rate in *MA* is significantly higher than that in *LA* (Mann-Whitney test, *MA* vs. *LA*, *p* < 0.0001).

**Figure 5:**
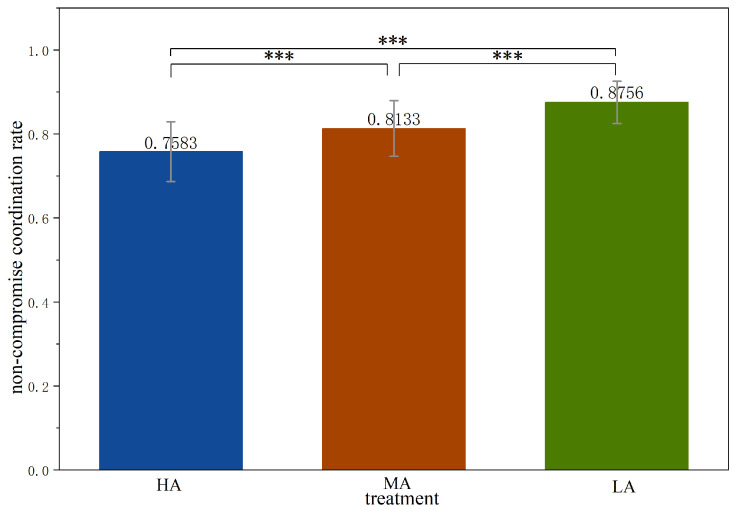
Average non-compromise coordination rate across treatments. Notes: *** p<0.01.

As shown in Figure 5, the non-compromise coordination rate is 0.7583 in *HA*, lower than in other treatments (0.8133 in *MA*, and 0.8756 in *LA*). The non-compromise coordination rate in *HA* is significantly lower than in other treatments (Mann-Whitney test, *HA* vs. *MA*, *p* < 0.0001; *HA* vs. *LA*, *p* < 0.0001). The non-compromise coordination rate in *MA* is also significantly lower than that in *LA* (Mann-Whitney test, *MA* vs. *LA*, *p* < 0.0001). The above findings are summarized in the following results.

**Result** **2.** *The frequencies of compromise outcomes across the three treatments are such that HA>MA>LA. The frequencies of non-compromise coordination outcomes across the treatments have the opposite order, i.e., HA<MA<LA.*

In our experiment, equilibrium payoffs for the unfavorable option in *HA* and *MA* are both lower than for the compromise option (50 vs. 100 and 80 vs. 100, respectively). Compared to the lower payoff of the unfavorable option, subjects in *HA* and *MA* prefer the compromise outcome. In contrast, in *LA*, equilibrium payoff at the unfavorable option is higher than the compromise option (120 vs. 100), leading to both significantly lower compromise rate and significantly higher non-compromise coordination rate compared to the other two treatments. Figure 4 and Figure 5 present these results at the treatment level, and the detailed results for each phase are reported in Appendix C.

#### 4.1.2. The Effect of Payoff Asymmetry on Strategy Adoption

**Figure 6:**
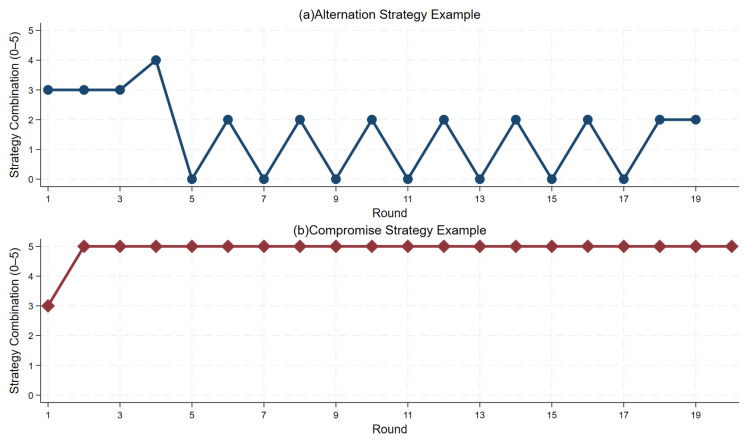
Examples of alternation strategy and compromise strategy. *Notes:* The x-axis denotes the round number, and the y-axis reports the distribution of outcomes. Values from 0 to 5 correspond to the following action profiles: 0 = (A, A); 1 = (A, B) or (B, A); 2 = (B, B); 3 = (A, C) or (C, A); 4 = (B, C) or (C, B); 5 = (C, C).

The previous section studies the coordination outcome, defined as an outcome in which both subjects in a pair receive positive payoffs in a given round. We now investigate coordination strategies subjects adopt over multiple rounds, which may be dynamically changing (for example, the alternation strategy). We classify subjects’ strategies in rounds 11–20 of each phase as follows: if subjects and their partners alternate consistently between options A and B for more than four rounds, we consider them to have adopted a stable alternation strategy, as shown in Figure 6a; if they choose compromise option C consistently for more than four rounds, we consider them to have adopted a stable compromise strategy, as shown in Figure 6b. If neither condition is met, we classify behavior patterns as other strategies.

The reason we focus on rounds 11–20 is that subjects are randomly rematched at the start of each phase, and their strategies depend on their expectations about the new partner. Playing in a new match needs adjustment time, even for experienced subjects. Early rounds mainly capture subjects adjusting to each other, while later rounds better reflect stable strategy choices. Appendix C reports robustness checks using rounds 1–20, 3–20, 5–20, 7–20, and 9–20, confirming our findings in the main text.

**Figure 7:**
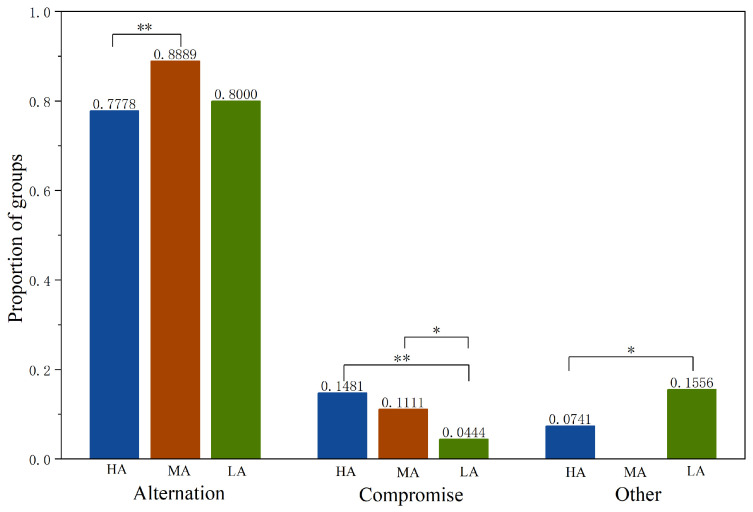
Proportion of groups adopting each strategy across treatments. Notes: ** p<0.05, * p<0.1.

Now we investigate whether the adoption of the compromise strategy and alternation strategy differs across treatments, as shown in Figure 7.^4^ The adoption of the compromise strategy in *LA* is lower than in *HA* and *MA* (Mann-Whitney, *HA* vs. *LA*, *p* = 0.0162; *MA* vs. *LA*, *p* = 0.0959), while the difference between *HA* and *MA* is not significant (Mann-Whitney test, *HA* vs. *MA*, *p* = 0.4434). The adoption of the alternation strategy in *HA* is lower than in *MA* (Mann-Whitney test, *HA* vs. *MA*, *p* = 0.0395), while the difference between *HA* and *LA* or *MA* and *LA* is not significant (Mann-Whitney test, *HA* vs. *LA*, *p* = 0.7040; *MA* vs. *LA*, *p* = 0.1009).

We report the above finding as Result 3. The proportion of groups adopting each strategy across treatments in each phase is reported in Appendix C.

**Result** **3.** *The frequencies of the compromise strategy adopted in HA and MA are higher than in LA, while the frequency of the alternation strategy adopted in HA is higher than in MA.*

**Figure 8:**
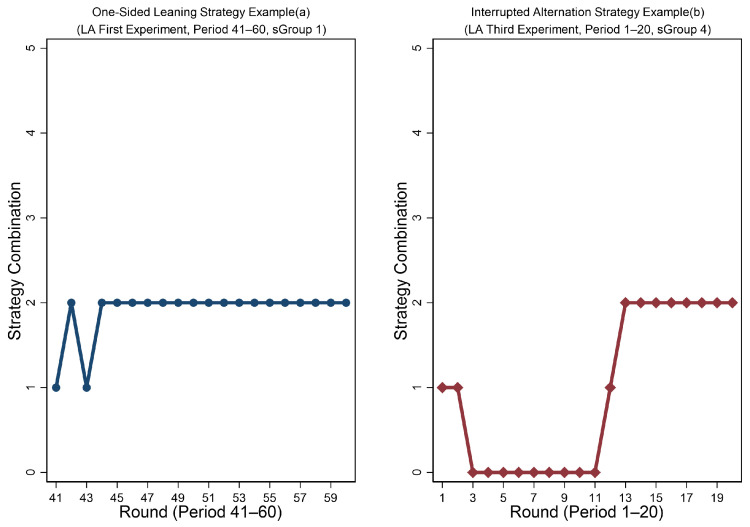
Examples of “one-sided” strategy and “interrupted alternation” strategy.

The pattern of the subjects’ coordination strategy reported in Result 3, which is mainly consistent with the pattern of the pair’s coordination outcome reported in Result 2. These findings naturally raise a question: why is the compromise strategy adopted less frequently in *LA*? The degree of payoff asymmetry does not appear to fully explain this pattern. We observe that in *LA*, two additional strategies appear that are rarely seen in *HA* and *MA*. The first is the “one-sided” strategy, where both subjects almost always choose the same subject’s favorable option, as illustrated in Figure 8a. The second is the “interrupted alternation” strategy. Within the 20 rounds of a phase, subjects first coordinate on one subject’s favorable option. Then, around rounds 11–13, the lower-payoff subject switches to their own favorable option. After 1–2 rounds of miscoordination, the other subject also switches to the first subject’s favorable option, as shown in Figure 8b.

The “one-sided” strategy and the “interrupted alternation” strategy in *LA* arise for two reasons. First, the payoff difference between favorable and unfavorable options is relatively small (120 vs. 180), enabling the lower-payoff subject to endure repeated disadvantage. Second, payoff from the unfavorable option remains higher than the compromise option (120 vs. 100), providing additional incentive to continue selecting the unfavorable option rather than switching to the compromise strategy. Therefore, these factors account for the observed non-alternation, non-compromise coordination patterns.

Overall, Hypothesis 1 is partially supported. Additionally, Figure 7 shows that the alternation strategy is the most frequently adopted strategy by subjects across all treatments. Therefore, in the next section, we focus on the adoption of alternation strategy.

### 4.2. The Effect of the Experience on the Adoption of the Alternation Strategy

This section studies the adoption of the alternation strategy. Unlike the compromise strategy, in which both subjects repeatedly choose the compromise option for more than 4 rounds, the alternation strategy requires subjects to take turns choosing their favorable and unfavorable options. In terms of coordination difficulty, the alternation strategy is harder to coordinate than the compromise strategy. First, we analyze how subjects’ prior experience with alternation affects their alternation strategy adoption rate. Second, we examine the alternation strategy adoption speed across phases and treatments.

We compare whether early alternation experience influences later alternation strategy adoption. The criterion for early alternation experience is defined as follows: a subject is considered to have early alternation experience if the subject and their partner achieved at least one successful alternation in any earlier phase. A double-experience pair refers to two subjects who both have early alternation experience, whereas a double-no-experience pair consists of two subjects who both lack such experience. A mixed pair is one in which only one partner has early alternation experience while the other does not.

**Figure 9:**
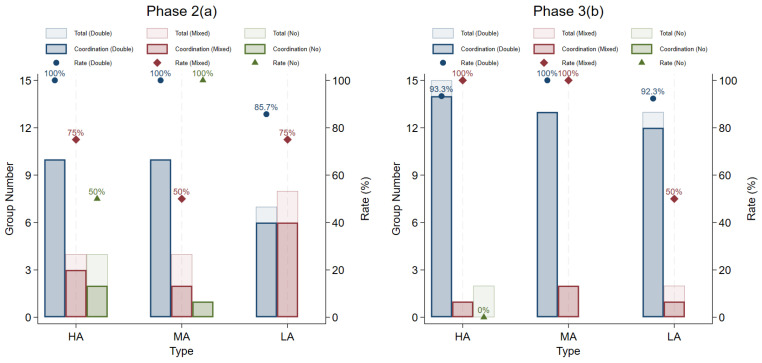
Experience of pairs and the rate of adopting alternation strategies in Phases 2–3.

Figure 9, uses light blue, light red, and light green to indicate the total number of double-experience pairs, mixed-experience pair and double-no-experience pairs; and dark blue, dark red, and dark green to indicate the number of pairs that successfully adopted the alternation strategy. Pairs with “double experience” adopt alternation strategies at a higher rate than “double no experience” (Mann-Whitney test, *p* = 0.0118 in *Phase 2*; *p* < 0.001 in *Phase 3*) or “mixed” pairs (Mann-Whitney test, *p* = 0.0128 in *Phase 2*; *p* = 0.2010 in *Phase 3*). Pairs with “mixed experience” tend to adopt alternation strategies more often than “double no experience” (Mann-Whitney test, *p* = 0.0723 in *Phase 2*; *p* = 0.0736 in *Phase 3*).

These differences suggest that prior alternation experience plays a key role in alternation strategy adoption: On the one hand, prior alternation experience makes subjects more familiar with the strategy and improves coordination in later phases. On the other hand, in double-experience pairs, both subjects have prior experience, which makes coordination easier and reduces the number of trial-and-error rounds. In mixed-experience pairs, only one subject has prior experience, increasing coordination difficulty, so their adoption rate falls between double-experience and double-no-experience pairs. Double-no-experience pairs must rely on repeated interactions to achieve alternation, leading to a lower adoption rate of the alternation strategy. We report the above findings as Result 4.

**Result** **4.** *The adoption rate of alternation strategies is highest in double-experience pairs, followed by mixed-experience pairs, and lowest in double-no-experience pairs.*

From Result 4, we find that prior successful alternation experience increases the likelihood that subjects adopt the alternation strategy. Next, we examine how payoff asymmetry affects alternation strategy adoption speed across phases. The adoption speed of the alternation strategy is defined as follows: first, it is measured over rounds 1–20 of each phase for any given treatment. Second, for all pairs at a given phase within the same treatment that adopt the alternation strategy, we record the number of rounds it takes before the first adoption of the alternation strategy. Finally, we sum up these numbers of rounds across adopting pairs and divide by the number of adopting pairs. The average number of rounds defines the adoption speed at the given phase for the given treatment.

**Figure 10:**
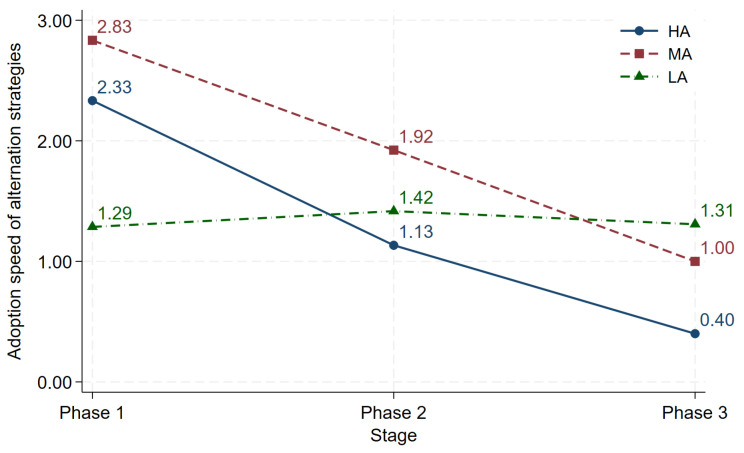
Adoption speed of the alternation strategy over phases across treatments.

From Figure 10, we find that in Phase 1, *LA* shows the fastest adoption, followed by *HA*, with *MA* showing the slowest adoption (Kruskal-Wallis test, *p* = 0.0122). This may occur because a larger payoff gap between the favorable option and the unfavorable option makes early coordination on an alternation pattern more difficult, and the payoff gap of *MA* is neither small enough for subjects to accept easily nor large enough to clearly motivate them to adopt the alternation strategy, which slows down its formation. In Phase 2, as subjects become more familiar with the strategy, adoption speeds across *HA*, *MA*, and *LA* do not differ significantly (Kruskal-Wallis test, *p* = 0.6782). While there are some differences, these are not statistically significant. A similar pattern is observed in Phase 3, where differences in adoption speed across payoff asymmetry levels are also not statistically significant (Kruskal-Wallis test, *p* = 0.1091). A possible explanation is that, with the accumulation of experience in *Phase 1* and *Phase 2* payoff asymmetry no longer affects the speed at which they adopt stable alternation strategies.

Figure 10 shows that a faster improvement in strategy adoption over time in *HA*, indicating that higher payoff asymmetry encourages subjects to adjust more quickly and reach stable patterns sooner (sign-rank test, *Phase 1* vs. *Phase 2*, *p* = 0.0088; *Phase 1* vs. *Phase 3*, *p* = 0.0003; *Phase 2* vs. *Phase 3*, *p* = 0.0147). The *MA* treatment shows a similar pattern to *HA*. (sign-rank test, *Phase 1* vs. *Phase 2*, *p* = 0.2043; *Phase 1* vs. *Phase 3*, *p* = 0.0333; *Phase 2* vs. *Phase 3*, *p* = 0.0424). In contrast, in *LA*, strategy adoption speed does not differ significantly across phases, suggesting that when payoff asymmetry is relatively lower, subjects’ coordination processes remain stable (sign-rank test, *Phase 1* vs. *Phase 2*, *p* = 0.5439; *Phase 1* vs. *Phase 3*, *p* = 0.5209; *Phase 2* vs. *Phase 3*, *p* = 0.9587). We report the above as Result 5. According to Results 4 and 5, Hypothesis 2 is supported.

**Result** **5.** *(a) Cross-treatment comparison: In Phase 1, the adoption speed of the alternation strategy is faster in LA than in HA and MA; in Phases 2 and 3, there is no significant difference in the adoption speed across treatments. (b) Over-phase comparison: In HA and MA, the adoption speed of the alternation strategy increases over phases; in LA, there is no significant difference in the adoption speed over phases.*

### 4.3. Efficiency and Fairness

#### 4.3.1. The Effect of Alternation and Compromise Strategies on Efficiency

In this section, we measure efficiency by the average payoff of subjects. Table 2 provides the average payoffs by type of strategies in each treatment. Across all treatments, average payoffs under the alternation strategy are significantly higher than those under the compromise and other strategies (Mann-Whitney test, *p* < 0.1 for all comparisons), as the compromise option (C, C) provides equal payoffs to subjects but yields a lower payoff than (A, A) or (B, B).

Results in Table 3 show that average payoffs increase as payoff asymmetry decreases. The average payoff in *LA* is significantly higher than in *HA* (Mann-Whitney test, *HA* vs. *LA*, *p* = 0.0779), whereas the differences in average payoffs between *HA* and *MA* and between *MA* and *LA* are not statistically significant (Mann-Whitney test, *HA* vs. *MA*, *p* = 0.3659; *MA* vs. *LA*, *p* = 0.3900).

There is an increase in the average payoff across phases. Average payoffs in *HA* increase significantly with the phases (sign-rank test, *p* < 0.01 for all pairwise comparisons). Specifically, the average payoff increases by 9.21% from Phase 1 to Phase 2 and by 8.88% from Phase 2 to Phase 3, suggesting a diminishing rate of growth across phases. In *MA*, the average payoff in Phase 3 is significantly higher than in the first two phases (sign-rank test, *Phase 1* vs. *Phase 2*, *p* = 0.1656; *Phase 2* vs. *Phase 3*, *p* = 0.0018; *Phase 1* vs. *Phase 3*, *p* = 0.0012). In *LA*, although the average payoff in the third phase also exceeds that in the first two phases, the differences are not statistically significant. We summarize the above findings on efficiency in the following result.

**Result** **6.** *The asymmetry of the payoff structure is negatively correlated with average payoffs. The learning effect due to repeated interactions contributes to the improvement of the average payoffs.*

#### 4.3.2. The Effect of Alternation and Compromise Strategies on Fairness

Fairness is measured by the difference in average payoffs between two subjects within the same phase. As shown in Table 4, payoff differences under other strategies are significantly higher than those under alternation and compromise strategies (Mann-Whitney test, *p* < 0.05 for all pairwise comparisons). In *HA* and *MA*, the difference between alternation and compromise strategies is not significant (Mann-Whitney test, *1.90* vs. *1.25*, *p* = 0.5342; *1.05* vs. *0*, *p* = 0.1908), suggesting that coordination through alternation and compromise enhances fairness.

As shown in Table 5, in *HA*, the payoff difference is largest in Phase 1, as this treatment features the highest level of payoff asymmetry and stable coordination has not yet been adopted in early interactions. In Phases 2 and 3, the payoff difference decreases significantly (sign-rank test, *12.78* vs. *2.78*, *p* = 0.2904; *2.78* vs. *0.56*, *p* = 0.0114; *12.78* vs. *0.56*, *p* = 0.0069). Specifically, the payoff difference decreases by 78.26% from Phase 1 to Phase 2 and by 80% from Phase 2 to Phase 3, indicating a faster reduction in payoff differences across later phases. In *MA* and *LA*, the payoff differences across different phases are not significant (sign-rank test, *p* > 0.1 for all pairwise comparisons), but within each treatment, the differences do not change substantially across phases. The payoff differences in *LA* are consistently higher than those in *MA*. Combining the above findings, since subjects in *MA* eventually coordinated on either alternation or compromise, their payoff differences are relatively small. In contrast, due to the low degree of payoff asymmetry in *LA*, subjects have a weaker incentive to learn to coordinate on an alternation strategy.

**Result** **7.** *The alternation strategy improves efficiency, while both the alternation and compromise strategies promote fairness.*

The results in Table 3 and Table 4 support Hypothesis 3. Under the alternation strategy, subjects take turns receiving favorable and unfavorable payoffs. Under the compromise strategy, subjects receive equal payoffs. Even in the presence of payoff asymmetry, both alternation and compromise strategies can enhance the fairness of payoff distribution.

## 5. Conclusions

This study investigates how payoff asymmetry influences coordination, efficiency, and fairness in a battle-of-the-sexes game with a compromise option. Our analysis yields four key findings. First, the degree of payoff asymmetry determines coordination outcomes. The highest asymmetry leads to the lowest coordination rates and increases the selection of the compromise option, while low asymmetry reduces the reliance on compromise, which is the risk-dominant strategy. Second, although the frequency of the compromise strategy adopted differs across asymmetry levels, the alternation strategy is the most frequently adopted coordination mode under all conditions. Third, in the dynamic analysis, we find that “double experience,” when both subjects have prior experience with alternation, increases the likelihood that they adopt alternation. In addition, higher payoff asymmetry increases learning, leading to faster growth in the adoption speed of alternation strategies across phases. Finally, we find a negative relationship between payoff asymmetry and average earnings, showing that structural inequality reduces efficiency even when coordination devices are available.

This study re-examines the compromise effect established by He and Wu (2020). While they demonstrate the option’s utility under varying matching protocols, we isolate payoff asymmetry as a key driver of strategy selection. Our results reveal that in spite of various degrees of payoff asymmetry, dynamic alternation prevails as the dominant long-term strategy across all conditions. Accumulated experience helps shape subsequent strategy adoption, and higher payoff asymmetry strengthens the learning effect. However, this coordination is achieved at a cost: we observe a structural efficiency loss, evidenced by the inverse relationship between the degree of asymmetry and average earnings.

Our results show that the role of the compromise option differs across treatments. Under high-conflict conditions (treatment *HA*), it plays an important role in helping subjects avoid unfair conflicts arising from large payoff gaps. Under low-conflict conditions (treatment *LA*), its role is relatively limited. For mechanism design, this suggests that introducing a compromise option is vital when conflicts of interest are severe, whereas facilitating stable partnerships is sufficient for groups to evolve toward mutually beneficial alternating patterns in low-conflict settings.

Although our experimental design advances the understanding of coordination under asymmetry, we draw our findings from a controlled laboratory setting using a specific game form with discrete strategy spaces. We implement a simplified matching protocol and use homogeneous participant pools, making small-stakes decisions. In future research, it would be interesting to see if our findings can be generalized to individuals with heterogeneous backgrounds, who are called upon to make high-stakes coordination decisions. It would also be interesting to see if introducing voluntary payoff redistribution options with different allocation rules would sustain or even enhance coordination levels in the battle-of-the-sexes game.

## Figures and Tables

**Table 1:** Summary of subjects.

Treatments	No. of Subjects	No. of Groups	No. of Sessions
HA	36	18	3
MA	30	15	3
LA	30	15	3
Total	96	48	9

**Table 2:** Payoff by type of strategies across treatments.

Treatment	Alternation	Compromise	Other
HA	141.4286	76.5625	53.7500
	(12.1381)	(22.7647)	(74.5582)
	[N = 84]	[N = 16]	[N = 8]
MA	137.2500	86.0000	–
	(20.1001)	(10.7497)	–
	[N = 80]	[N = 10]	–
LA	143.7500	50.0000	102.8571
	(10.9245)	(20.2814)	(47.0889)
	[N = 72]	[N = 4]	[N = 14]

Notes: Standard deviations are in parentheses; numbers of observations are in square brackets.

**Table 3:** Average payoffs over phases across treatments.

Treatment	Average	Phase 1	Phase 2	Phase 3
HA	125.3241	114.5833	125.1389	136.2500
	(38.6364)	(44.8191)	(39.7894)	(27.0218)
	[N = 108]	[N = 36]	[N = 36]	[N = 36]
MA	131.5556	122.3333	129.8333	142.5000
	(25.1522)	(30.3773)	(24.0403)	(15.0281)
	[N = 90]	[N = 30]	[N = 30]	[N = 30]
LA	133.2222	134.1667	124.5000	141.0000
	(31.2880)	(31.2013)	(40.3859)	(16.0495)
	[N = 90]	[N = 30]	[N = 30]	[N = 30]

Notes: Standard deviations are in parentheses; numbers of observations are in square brackets.

**Table 4:** The difference in average payoffs by type of strategies across treatments.

Treatment	Alternation	Compromise	Other
HA	1.9048	1.2500	50.0000
	(3.9504)	(3.4157)	(80.3564)
	[N = 84]	[N = 16]	[N = 8]
MA	1.0500	0.0000	–
	(2.5153)	(0.0000)	–
	[N = 80]	[N = 10]	–
LA	0.6667	3.0000	30.8571
	(1.2560)	(0.0000)	(21.3572)
	[N = 72]	[N = 4]	[N = 14]

Notes: Standard deviations are in parentheses; numbers of observations are in square brackets.

**Table 5:** The difference in average payoffs over phases across treatments.

Treatment	Phase 1	Phase 2	Phase 3
HA	12.7778	2.7778	0.5556
	(41.3771)	(4.5426)	(2.3231)
	[N = 36]	[N = 36]	[N = 36]
MA	0.9333	1.4000	0.4667
	(2.4202)	(2.8479)	(1.7760)
	[N = 30]	[N = 30]	[N = 30]
LA	5.8000	3.0000	7.6000
	(13.7123)	(5.8956)	(18.5391)
	[N = 30]	[N = 30]	[N = 30]

Notes: Standard deviations are in parentheses; numbers of observations are in square brackets.

**Figure A1:**
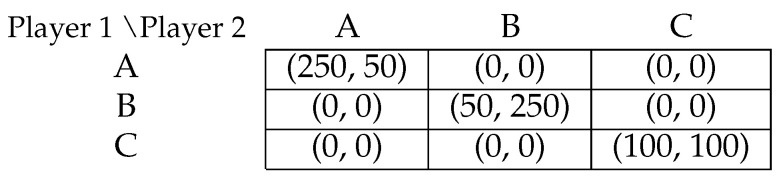
Payoff Matrix (ECU) of *HA*.

**Figure A2:**
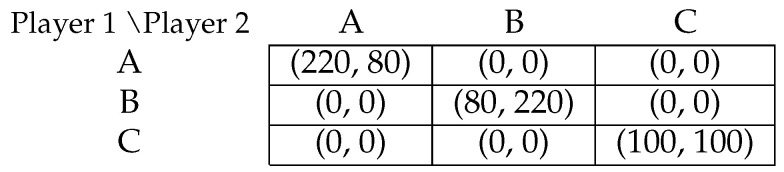
Payoff Matrix (ECU) of *MA*.

**Figure A3:**
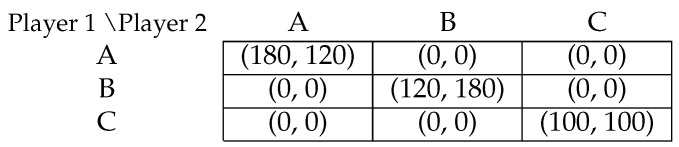
Payoff Matrix (ECU) of *LA*.

**Figure A4:**
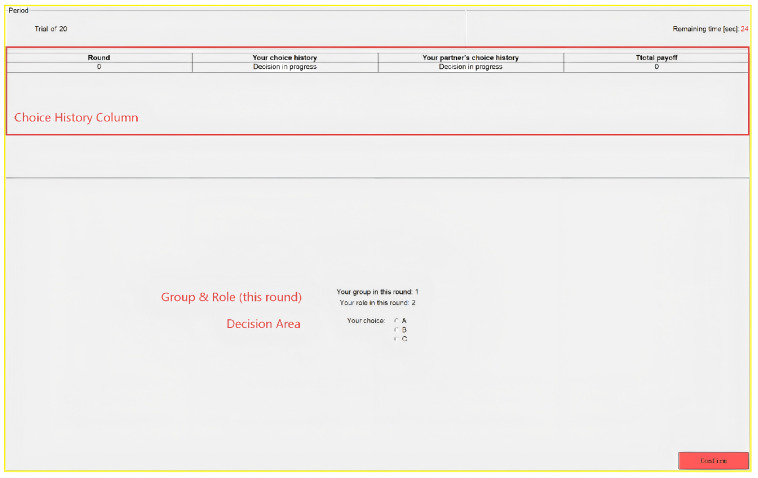
Decision interface (the interface through which participants make their choices).

**Figure A5:**
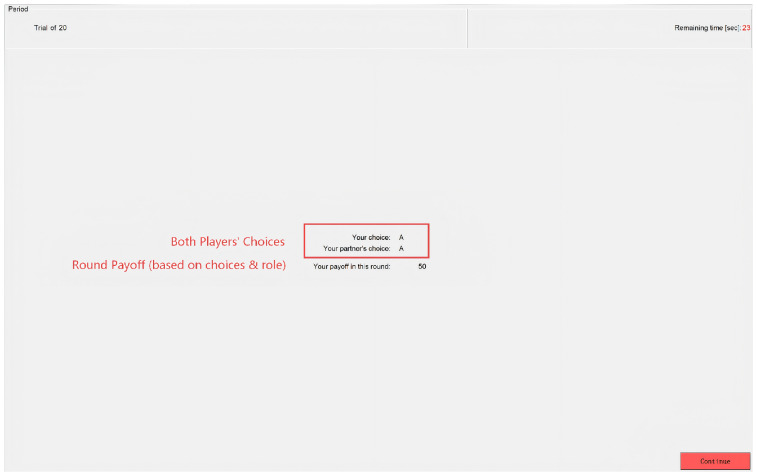
Payoff display interface (the interface showing participants’ earnings).

**Figure A6:**
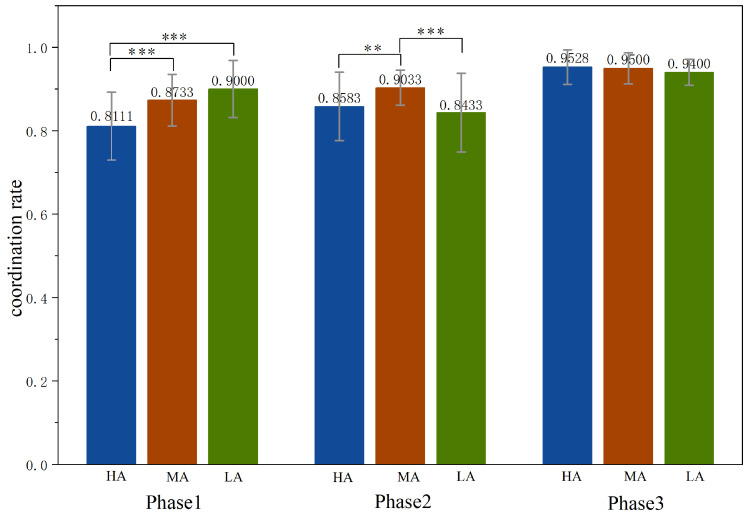
Average coordination rate across treatments in each phase. Notes: *** p<0.01, ** p<0.05.

**Figure A7:**
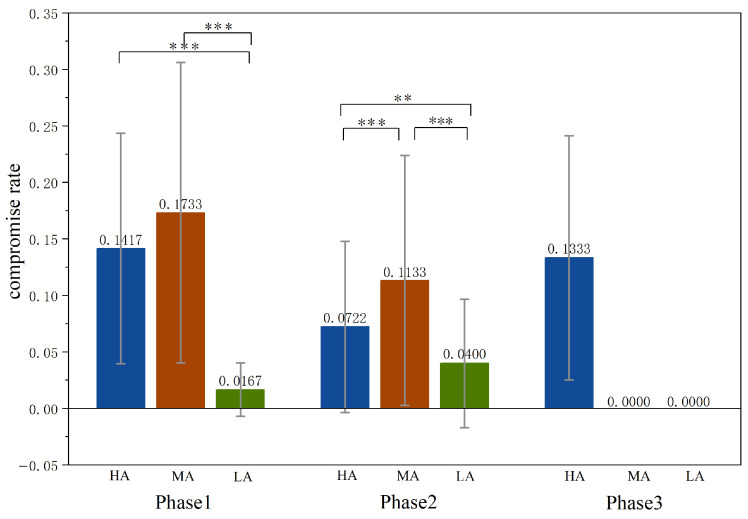
Average compromise rate across treatments in each phase. Notes: *** p<0.01, ** p<0.05.

**Figure A8:**
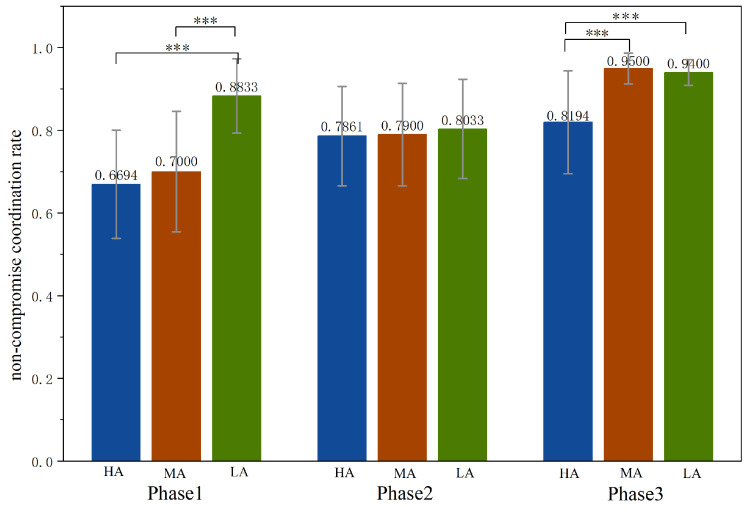
Average non-compromise coordination rate across treatments in each phase. *Notes:* *** p<0.01.

**Figure A9:**
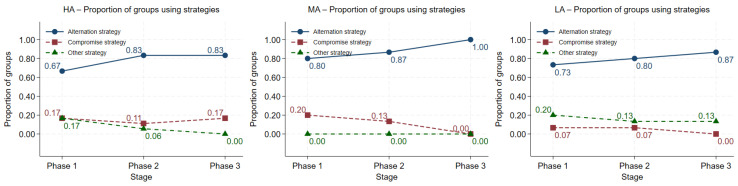
Proportion of groups choosing each strategy across treatments (11–20).

**Figure A10:**
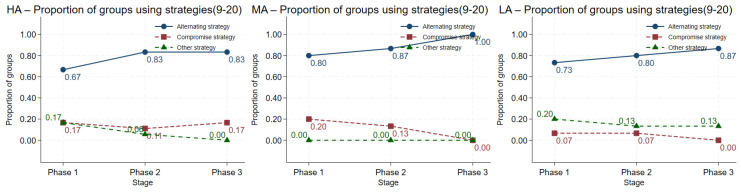
Proportion of groups choosing each strategy across treatments (9–20).

**Figure A11:**
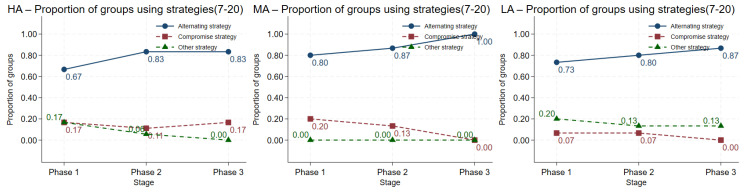
Proportion of groups choosing each strategy across treatments (7–20).

**Figure A12:**
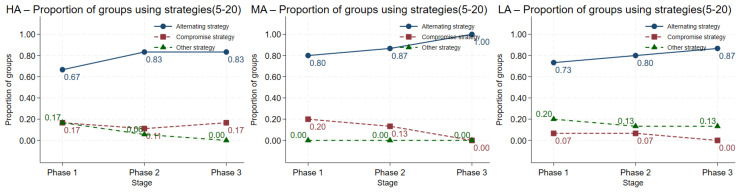
Proportion of groups choosing each strategy across treatments (5–20).

**Figure A13:**
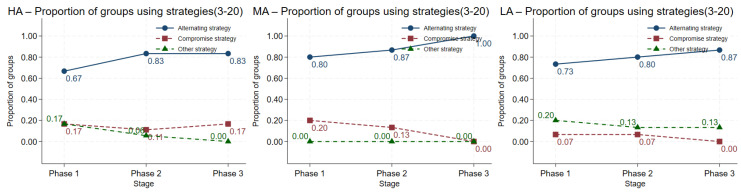
Proportion of groups choosing each strategy across treatments (3–20).

**Figure A14:**
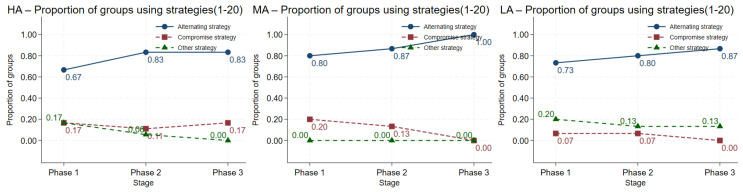
Proportion of groups choosing each strategy across treatments (1–20).

## Data Availability

The datasets generated and analyzed in the current study have been provided to the journal. They are not publicly available due to proprietary restrictions and their planned use in ongoing subsequent research, but are available from the corresponding author on reasonable request.

## Notes

1 Note that this game also has multiple mixed-strategy Nash equilibria. For a theoretical analysis on the mixed-strategy equilibria under the payoff structures we study, please see Appendix B.

2 These four phases consist of a practice phase (Phase 0) and three incentivized phases. For data analysis, we focus on the incentivized games, including Phase 1, Phase 2, and Phase 3.

3 The Mann–Whitney tests are not performed on the average rates in Figure 3, Figure 4 and Figure 5. For the average coordination rate, we construct a binary indicator that equals one if both players obtain positive payoffs and zero otherwise. The mean of this indicator corresponds to the coordination rate in Figure 3. The same procedure is applied to Figure 4 and Figure 5.

4 The Mann-Whitney tests are not performed on the proportions in Figure 7. Instead, for each pair we construct a binary indicator that equals one if the pair adopts the alternation strategy and zero otherwise. The mean of this indicator corresponds to the proportion of groups adopting the alternation strategy. The same procedure is applied to the compromise and other strategies.

## Appendix A. Experimental Instructions (Translated from Chinese)

Thank you for your participation in this experiment! Please read the following instructions carefully. If you have any questions, please feel free to ask us. Please note that you cannot communicate with other participants during the experiment. You will be paid to complete the experiment according to the instructions. Your earnings from the experiment will be determined by your choices and the choices of other participants. At the end of the experiment, the tokens that you have earned will be converted into CNY at the exchange rate of 400 tokens = 1 CNY.

### Appendix A.1. Matching Rules [Common to All Treatments]

The experiment consists of one practice phase and three main phases, each comprising 20 rounds of decision-making. The rules and experimental settings in the practice phase are identical to those in the main phases; however, outcomes from the practice phase will not count toward your final earnings.

At the start of each phase, you will be randomly matched with another participant to form a two-person group. Within each matched pair, participants will be randomly assigned the roles of Player 1 and Player 2. You two will then complete 20 rounds of decision-making under these assigned roles.

Group formation and role assignment are independently redetermined at the beginning of each new phase. This means you will be matched with different participants across phases, with each phase’s grouping being completely independent. However, within a given phase, your group composition and assigned role will remain unchanged throughout all 20 rounds.

### Appendix A.2. Playing Rules

In each round, you and your matched participant will simultaneously choose among three available actions: A, B, and C. The payoffs for both players (denominated in Experimental Currency Units, ECU) are jointly determined by the action profile selected.

[Specific to the treatment *HA*]

The specific payoff structure is defined as follows:

1. If both players choose action *A*, Player 1 receives 250 ECU and Player 2 receives 50 ECU.
2. If both players choose action *B*, Player 1 receives 50 ECU and Player 2 receives 250 ECU.
3. If both players choose action *C*, each player receives 100 ECU.
4. If the players choose different actions, both receive 0 ECU.

The payoff structure is presented in Figure A1 (ECU), where columns correspond to Player 1’s actions and rows correspond to Player 2’s actions.

[Specific to the treatment *MA*]

The specific payoff structure is defined as follows:

1. If both players choose action *A*, Player 1 receives 220 ECU and Player 2 receives 80 ECU.
2. If both players choose action *B*, Player 1 receives 80 ECU and Player 2 receives 220 ECU.
3. If both players choose action *C*, each player receives 100 ECU.
4. If the players choose different actions, both receive 0 ECU.

The payoff structure is presented in Figure A2 (ECU), where columns correspond to Player 1’s actions and rows correspond to Player 2’s actions.

[Specific to the treatment *LA*]

The specific payoff structure is defined as follows:

1. If both players choose action *A*, Player 1 receives 180 ECU and Player 2 receives 120 ECU.
2. If both players choose action *B*, Player 1 receives 120 ECU and Player 2 receives 180 ECU.
3. If both players choose action *C*, each player receives 100 ECU.
4. If the players choose different actions, both receive 0 ECU.

The payoff structure is presented in Figure A3 (ECU), where columns correspond to Player 1’s actions and rows correspond to Player 2’s actions.

For each round of the play, it has two stages, which are the **Decision-Making Stage** and the **Payoff Display Stage**.

#### Appendix A.2.1. First Stage: Decision-Making Stage [Common to All the Treatments]

The decision-making interface is divided into two distinct sections. The upper section constitutes the History Record Panel, which displays the decision history and accumulated total earnings for you and your current partner throughout previous game phases. The lower section presents your current group composition and assigned player role (Player 1/Player 2) for this phase, along with three available action choices. You will make your decision for the current round by selecting among these three options and subsequently clicking the “Confirm” button in the lower-right corner to proceed to the next stage.

The decision interface is illustrated in the following figure:

#### Appendix A.2.2. Second Stage: Payoff Display Stage [Common to All the Treatments]

During the second stage, the interface displays the choices made by you and your matched participant in the current round, and presents the corresponding payoffs (ECU). After confirming the information, you may click the “Continue” button in the lower-right corner to proceed to the next decision-making round.

The payoff display interface is illustrated in the following figure:

### Appendix A.3. Payment Rules [Common to All the Treatments]

The accumulated tokens that you have earned across the three main game phases will be converted into CNY at the exchange rate of 400 tokens = CNY 1. We will take your converted earnings plus a 5 CNY show-up fee as your final payment in the experiment. The resulting amount will be paid to you via WeChat Pay immediately after the experiment is over.

If you have any questions, please contact the experimenter. If there is no problem, we will start the experiment after all participants confirm.

## Appendix B. Mixed-Strategy Nash Equilibrium

In this appendix, we characterize the Nash equilibria under the three payoff structures: High Asymmetry (*HA*), Moderate Asymmetry (*MA*), and Low Asymmetry (*LA*). In all treatments, the game admits three pure-strategy Nash equilibria: two efficient but asymmetric coordination outcomes, (A, A) and (B, B), and one inefficient symmetric compromise outcome, (C, C).

Beyond pure strategies, the game admits multiple mixed-strategy equilibria. We characterize equilibria where players mix over a subset of two actions (Partially Mixed) and equilibria where players mix over all three actions (Fully Mixed).

Table A1 summarizes the probability distributions for these equilibria. For notation, we define the mixed strategy of player *i* as $\sigma_{i}=\left(p_{fav},p_{unfav},p_{comp}\right)$, where $p_{fav}$, $p_{unfav}$, and $p_{comp}$ represent the probabilities of choosing the payoff-favorable action, the unfavorable action, and the compromise option, respectively. Due to the symmetry of the game structure, the equilibrium strategies are symmetric in terms of these positions.

Table A1 also illustrates the comparative statics of the mixed-strategy equilibrium. As payoff asymmetry decreases (from *HA* to *LA*), the probability of choosing the preferred action in the fully mixed equilibrium declines (0.588→0.349), while the probability of choosing the compromise option increases (0.294→0.419). This implies that if subjects were adopting the fully mixed strategies, the compromise option should be observed more frequently in the data as asymmetry diminishes, a prediction that stands in contrast to the behavioral patterns observed in our experiment. It is also worth noting that this comparative statics result is not robust when subjects adopt a partially mixed strategy between the unfavorable option and the compromise option. Since the play of mixed strategies is not that intuitive and the existence of multiple mixed-strategy equilibria makes it technically challenging to analyze, we restrict our attention to the analysis of pure strategies in the main text.

**Table A1:** Mixed-Strategy Nash Equilibria across Treatments.

Treatment	Equilibrium Support	$p_{\mathrm{fav}}$	$p_{\mathrm{unfav}}$	$p_{\mathrm{comp}}$
1. High Asymmetry (*HA*)				
Partially Mixed	{Fav, Unfav}	$\frac{5}{6}\approx 0.8333$	$\frac{1}{6}\approx 0.1667$	0
	{Fav, Comp}	$\frac{2}{3}\approx 0.6667$	0	$\frac{1}{3}\approx 0.3333$
	{Unfav, Comp}	0	$\frac{2}{7}\approx 0.2857$	$\frac{5}{7}\approx 0.7143$
Fully Mixed	{All Actions}	$\frac{10}{17}\approx 0.5882$	$\frac{2}{17}\approx 0.1176$	$\frac{5}{17}\approx 0.2941$
2. Moderate Asymmetry (*MA*)				
Partially Mixed	{Fav, Unfav}	$\frac{11}{15}\approx 0.7333$	$\frac{4}{15}\approx 0.2667$	0
	{Fav, Comp}	$\frac{5}{9}\approx 0.5556$	0	$\frac{4}{9}\approx 0.4444$
	{Unfav, Comp}	0	$\frac{5}{16}\approx 0.3125$	$\frac{11}{16}\approx 0.6875$
Fully Mixed	{All Actions}	$\frac{55}{119}\approx 0.4622$	$\frac{20}{119}\approx 0.1681$	$\frac{44}{119}\approx 0.3697$
3. Low Asymmetry (*LA*)				
Partially Mixed	{Fav, Unfav}	$\frac{3}{5}\approx 0.6000$	$\frac{2}{5}\approx 0.4000$	0
	{Fav, Comp}	$\frac{5}{11}\approx 0.4545$	0	$\frac{6}{11}\approx 0.5455$
	{Unfav, Comp}	0	$\frac{5}{14}\approx 0.3571$	$\frac{9}{14}\approx 0.6429$
Fully Mixed	{All Actions}	$\frac{15}{43}\approx 0.3488$	$\frac{10}{43}\approx 0.2326$	$\frac{18}{43}\approx 0.4186$

Notes: *p_fav_*, *p_unfav_*, and *p_comp_* denote the probability that a player chooses their payoff-favorable option (e.g., A for Player 1), their unfavorable option (e.g., B for Player 1), and the compromise option C, respectively.

## Appendix C. Supplemental Figures

In this appendix, we provide supplemental figures to help understand the experimental results and assess the robustness of the strategies. Figure A6, Figure A7 and Figure A8 show the average coordination rate across treatments in each phase.

Figure A9 show the proportion of groups that choose each strategy across treatments in each phase. Figure A10, Figure A11, Figure A12, Figure A13 and Figure A14 show the proportion of groups that choose each strategy across treatments in each phase after relaxing the restrictions in rounds 11–20. In Figure A10, Figure A11, Figure A12, Figure A13 and Figure A14, we recalculated the results using rounds 9–20, 7–20, 5–20, 3–20, and 1–20, respectively.
